# Promoting community knowledge and action for malaria control in rural Cambodia: potential contributions of Village Malaria Workers

**DOI:** 10.1186/1756-0500-5-405

**Published:** 2012-08-03

**Authors:** Sachiko Lim, Junko Yasuoka, Krishna C Poudel, Po Ly, Chea Nguon, Masamine Jimba

**Affiliations:** 1 Department of Community and Global Health, Graduate School of Medicine, University of Tokyo, 7-3-1 Hongo, Bunkyo-ku, Tokyo, 113-0033, Japan; 2 The National Centre for Parasitology, Entomology and Malaria Control, 372 Monivong Boulevard, Phnom Penh, Cambodia

**Keywords:** Malaria, Village Malaria Workers, Treatment-seeking behavior, Knowledge, Preventive measures, Symptoms, Antimalarial drug, Community, Cambodia, Public health

## Abstract

**Background:**

Cambodia has been investing in Village Malaria Workers (VMWs) to improve malaria case management in rural areas. This study assessed the quality of the VMWs’ services compared to those by a government-run health center from the perspective of community members. We focused on VMWs’ contribution to promote their action to control malaria. A community-based cross-sectional study was conducted in Kampot province in 2009. Interviews were conducted at every accessible household in a village with VMWs (n = 153) and a village with a health center (n = 159), using interviewer administered questionnaire. Preference of the interview was given to female household head. Multiple regression analyses were run to compare knowledge about malaria, preventive measures taken, and time before first malaria treatment between the two villages.

**Findings:**

The villagers perceived the VMWs’ services equally as good as those provided by the health center. After controlling for confounding factors, the following indicators did not show any statistical significance between two villages: community members’ knowledge about malaria transmission (AOR = 0.60, 95% CI = 0.30-1.22) and government-recommended antimalarial (AOR = 0.55, 95% CI = 0.25-1.23), preventive measures taken (Beta = −0.191, p = 0.315), and time before the first treatment (Beta = 0.053, p = 0.721). However, knowledge about malaria symptoms was significantly lower in the village with VMWs than the village with a health center (AOR = 0.40, 95% CI = 0.19-0.83).

**Conclusions:**

VMWs played an equivalent role as the health center in promoting malaria knowledge, action, and effective case management. Although VMWs need to enhance community knowledge about malaria symptoms, the current government policy on VMWs is reasonable and should be expanded to other malaria endemic villages.

## Findings

### Background

Malaria continues to be a leading cause of morbidity and mortality worldwide, although the disease is preventable and treatable [1]. World Malaria Report 2011 estimated 216 million clinical cases and 655 000 malaria deaths worldwide in 2010 [1]. According to the most systematic assessment of mortality done by Murray CJ and colleagues, global malaria deaths was 1.24 million in 2010, which was almost double compared to World Malaria Report’s estimate [2]. Their estimate implies that malaria may impose even greater burden on populations living in endemic areas.

Globally, early diagnosis and prompt treatment with effective antimalarial drug are the cornerstones of current malaria control policy [1]. The success of this strategy at community-level depends on early recognition of the symptoms and the subsequent treatment-seeking behavior [3]. Many studies have found numerous factors associated with treatment-seeking behavior for malaria. They include affordability of treatment, availability and effectiveness of drugs, geographic accessibility, and perception of severity of the illness, quality of care and cultural beliefs about the cause [4]. Many of these factors are specific to the local context. An effective malaria control strategy, thus, requires accurate information on local perceptions and practices regarding malaria [5].

In many malaria-endemic countries, however, key challenges have been the lack of human capacity and health systems for delivering essential interventions [6]. The potential contribution of community health workers (CHWs) has renewed interest in overcoming these challenges, since they act as the first line of contact with the health system in most resource-poor countries [7]. A recent study from Zambia demonstrated that CHWs were able to manage malaria related fevers by correctly interpreting Rapid Diagnostic Test (RDT) results and appropriately prescribing antimalarials with parasitological confirmation [8]. Evidence from Ghana showed CHWs achieved higher coverage and adherence of intermittent preventive treatment of malaria in children [9].

In Cambodia, malaria continues to be a major public health problem. Over 6 million people are living in high transmission area [1]. Malaria incidence is particularly high in remote and resource-poor villages without public health services. The specific high-risk groups are pregnant women and children living in heavily forested villages, and forestry workers who have recently migrated from non-endemic areas [10]. In 2010, Cambodia had 49,356 probable and confirmed malaria cases and 151 malaria attributed deaths. *Plasmodium falciparum* accounted for 66% of confirmed malaria cases [1].

To improve malaria case management in remote forested areas, Cambodia has undertaken the Village Malaria Worker (VMW) project as part of its national malaria control program. The VMW project started in Rattanakiri province in northeastern Cambodia in 2001. A further pilot project was implemented in Koh Kong province in 2002. Based on the success of these pilot efforts, the VMW project was scaled up to cover 300 villages in seven provinces in 2004, with financial support from the Global Fund to Fight AIDs, Tuberculosis and Malaria. It has since been further scaled up to 1528 villages in 17 provinces (Cambodia National Malaria Center, Ministry of Health of the Kingdom of Cambodia, unpublished observations).

VMWs received two days of training under supervision of Cambodia National Malaria Center. Through the training, they learn cause, symptoms of malaria, and how to perform RDTs (*Plasmodium falciparum* only) on villagers suspected of having malaria and provide treatment according to national guidelines. The curriculum also includes identifying danger signs, and referring severe cases to the nearest health center, as well as recording and reporting the number of RDTs performed, malaria cases detected, and antimalarial used [11]. VMWs render services in their homes for free of charge. HC staff members have three years of nursing school training in providing basic treatment for a wide variety of diseases.

A previous study evaluated the nature and quality of the VMWs’ services by interviewing VMWs [12]. However, few studies have identified the similarities and differences between the quality of the VMWs’ services and those by a government-run health center from the perspective of community members.

This study, therefore, aimed at comparing the quality of services in promoting accurate knowledge about malaria, antimalarial, effective malaria control measures, and appropriate treatment-seeking behavior between a village with VMWs and a village with a health center. This study will provide important information for devising strategies to improve access to prompt and effective malaria treatment in underserved rural areas.

## Methods

### Study design and study area

A community-based cross-sectional study was conducted in Kampot province. The Province is one of the malaria-endemic areas in rural Cambodia. It is located about 150 km south of the capital, Phnom Penh, and had an estimated population of 620,000 in 2008 (Cambodia National Malaria Center, Ministry of Health of the Kingdom of Cambodia, unpublished observations). The province has 522 villages and four operational district hospitals (ODHs) and 49 health centers (HCs) (Cambodia National Malaria Center, Ministry of Health of the Kingdom of Cambodia, unpublished observations). Malaria is endemic in 133 villages of the villages. Of these villages, 27 have VMWs, who have been trained to perform rapid diagnostic tests on villagers suspected of having malaria and provide treatment according to national guidelines, and 84 have village health volunteers (VHVs), who have been trained to provide malaria-related health education in their communities. Most malaria transmission takes place during or shortly after the rainy season, with the peak between July and September.

Of 133 malaria endemic villages, we purposively selected one VMW village and one HC village based on similar characteristics of malaria incidence and population. A VMW village was defined as a village without a health center where VMWs are serving. On the other hand, a HC village was defined as a village where a health center is located but VMWs are not serving. Andeng Sang, the VMW village, and Srakak Neak, the HC village are 10 km apart. The VMW project was implemented in Andeng Sang in 2005. As is the case with other VMW villages, two VMWs were serving in the village. The Koh Slah Health Center in Srakak Neak is staffed by five nurses and four midwives; it is also the nearest health center to Andeng Sang. According to a 2008 survey by Cambodia’s National Malaria Center (CNM), Andeng Sang had 606 inhabitants and 55 reported malaria-positive cases and Srakak Neak had 651 inhabitants and 55 reported malaria-positive cases.

### Selection of study participants

We visited every accessible household (Andeng Sang: 153/165 households; Srakak Neak: 159/166 households) and screened members for inclusion in interviews. Twelve households in the VMW village and seven households in the HC village could not be reached due to fear of unexploded landmines or geographic inaccessibility. In each household, preference for the interview was given to the primary caregiver, usually the female head of the household. In her absence, the male head of the household was interviewed. If neither was present, a responsible adult above the age of 18 (e.g. mother/father/daughter/son of female/male household head), who understood family medical history and treatment-seeking behavior as well as care givers, was interviewed. Only one person per household was interviewed.

### Data collection

We collected data in August, 2009, during the peak malaria transmission season. An interviewer administered questionnaire was used to collect data after conducting pre-tests. Household interviews were conducted by eight trained field workers who spoke the local language, Khmer, and were fully familiar with the local culture and sensitivities. The questionnaire had been translated from English into Khmer, and the respondents’ answers were translated into English. Questionnaire administration was monitored daily for quality control.

The questionnaire addressed the following topics:

 (1) Socio-demographic characteristics of the respondent and his or her family.

 (2) Knowledge about malaria (its transmission route and symptoms, name of government-recommended antimalarial drug).

 (3) Actions taken to prevent malaria (personal preventive measures and mosquito control measures).

 (4) Treatment-seeking behavior for malaria (history of fever and malaria infection, symptoms that prompted patients to seek care outside the home, type and number of treatment sources consulted, reason for choosing the first provider, time from symptom onset to receiving the first treatment, type of diagnosis, and form of therapy given).

### Measures

Knowledge about malaria was measured in three parts: malaria transmission, malaria symptoms, and government-recommended antimalarial. Regarding transmission, respondents who answered that mosquito bites were the only route for transmission were given one point. Those who gave another cause or a number of causes in addition to mosquito bites were given zero points. Regarding malaria symptoms, this study defined fever, shivering, and sweating as the three major malaria symptoms. Respondents who named all three major symptoms were given one point. Those who gave other malaria symptoms in addition to the three major symptoms were also given one point. Those who couldn’t name all of three major symptoms were given zero points. Regarding government-recommended antimalarial, A + M was the first line antimalarial drug for uncomplicated malaria recommended by Cambodian government. Although Malarine was a commercialized antimalarial, it contains the same drug component as A + M. Thus, we also assumed Malarine as the recommended first line antimalarial drug. Those who gave correct name of A + M or Malarine were given one point. Those who gave the name of drug other than the above two were given zero point.

The number of personal preventive measures and mosquito control measures taken were calculated. Respondents who had taken personal measures with potential preventive effects (use of a treated mosquito net, wearing long sleeves and trousers, burning cow dung/leaves, using mosquito coils, using repellents, and closing windows and doors) were given one point. Respondents were given one point for each mosquito control measure taken (draining stagnant water and keeping the compound clean and/or building latrines to reduce mosquito breeding/resting places), regardless of the frequency. Total scores ranged from 0 to 9. Scores for the time between the onset of symptoms and the first malaria treatment were: same day = 0, next day = 1, two days after onset = 2, three or more days after onset = 3.

### Data analysis

Statistical analyses were performed using STATA version 11. The student *t*-test was used to compare age and number of household members in the VMW village and the HC village. Differences in proportions were compared for significance using the Chi-square test or Fisher’s exact test. To run multiple regression analyses, we assessed multicollinearity between 12 potential confounders. We assumed that multicollinearity exists when correlation coefficient was above 0.50. As a result, we excluded 3 variables which had collinearity with other variables. They were the nearest health facility, place of blood test and the number of children under 18 years. We selected the variables which seemed to have bigger impact on community knowledge, actions and treatment seeking behavior. We also included variables which were not statistically significant in bivariate analyses, but were found to be determinants of treatment-seeking behavior and malaria-related knowledge in the previous studies. We included them because insignificant variables in bivariate analysis may become significant in multivariate analysis. We finally included 9 potential confounders in the regression models. They were age, gender, marital status, highest level of education, occupation, number of household member, monthly income, first treatment source for malaria-like symptoms, and distance from health facility. Primary outcome variables in the multiple regression analyses were (1) knowledge about the cause of malaria, (2) knowledge about malaria symptoms, (3) knowledge about government-recommended antimalarial for uncomplicated malaria, (4) preventive and control measures taken, and (5) time before first malaria treatment. A *p*-value of <0.05 was considered statistically significant.

### Ethical considerations

Ethical approval was obtained from the Cambodian Ministry of Health and the Ethical Committee of the Faculty of Medicine at the University of Tokyo. Those who were eligible for the study were informed of the purpose and procedure of the study and were asked for their voluntary participation in the study. Individual written informed consent was obtained from all participants prior to interviews. Confidentiality was protected at all stages of the data analyses.

## Results

### Response rate

The interview response rate was 92.7% (153/165) in the VMW village and 95.8% (159/166) in the HC village. Socio-demographic characteristics of the respondents in the VMW village and the HC village are described in Table 1. Occupation, monthly income, first treatment source and distance from the health facility differed significantly between the two villages.

**Table 1:** Socio-demographic characteristics of the respondents

**Characterictics (n=312)**	**VMW village (n=153)**				**HC village (n=159)**				**p**
	**Means**	**SD**	**n**	**%**	**Means**	**SD**	**n**	**%**	
Age (years)*	37.8	13.3			38.4	13.7			0.696
**Gender**†									0.210
Male			41	26.8			33	20.7	
Female			112	73.2			126	79.3	
**Marital status**†									0.082
Married			117	76.5			134	84.3	
Not married			36	23.5			25	15.7	
**Highest level of education**†									0.173
Primary			70	45.8			89	56.0	
No education			60	39.2			48	30.2	
Secondary/High school			23	15.0			22	13.8	
**Occupation**‡									<.0001
Farmer			125	81.7			154	96.9	
Other plus none			28	18.3			5	3.1	
Number of household members*	4.8	2.1			4.9	1.9			0.765
**Monthly income (USD)**†									0.005
Monthly income<50			134	87.6			153	96.2	
50≤Monthly income			19	12.4			6	3.8	
**First treatment source**‡									<.0001
VMW			55	36.0			0	0	
HC			52	34.0			150	94.3	
Other health facilities			46	30.0			9	5.7	
**Distance from health facility**‡									<.0001
<2km			38	24.8			123	77.4	
2-5km			39	25.5			36	22.6	
>5km			76	49.7			0	0	

*Student *t-*test.

†Chi-square test.

‡Fisher’s exact test.

### Reported knowledge about malaria

The survey demonstrated the similar levels of knowledge on the cause of malaria symptoms, government-recommended antimalarial, and malaria risk populations between the VMW village and the HC village (Table 2). About 67% of total respondents had correct knowledge about the cause of malaria in both villages. More than 60% of total respondents correctly answered all of three major malaria symptoms in both villages. About 27% in the VMW village and 22% in the HC village gave the correct name of the government-recommended drug. Unlike children under age 5, only about 46% in the VMW village and 54% in the HC village perceived pregnant women as being vulnerable to malaria infection.

**Table 2:** Reported knowledge on malaria and action for malaria control

	**VMW village (n=153)**		**HC village (n=159)**		
	**n**	**%**	**n**	**%**	**p**
Knowledge on cause of malaria*†					0.937
Correct	102	66.7	106	67.1	
Wrong	51	33.3	52	32.9	
Knowledge on malaria symptoms*					0.802
Correct	98	64.1	104	65.4	
Wrong	55	36.0	55	34.6	
Name of government recommended antimalarial drug*					0.325
Correct	41	26.8	35	22.0	
Wrong	112	73.2	124	78.0	
**Knowledge on vulnerable groups to malaria infection**					
Under 5 years old*	109	71.2	118	74.2	0.556
Pregnant women*	70	45.8	85	53.5	0.173
**Personal protective and mosquito control measures**					0.574
No measures taken	8	5.2	13	8.2	
1 to 3	73	47.7	75	47.2	
4 to 6	72	47.1	71	44.7	

*Chi-square test.

†1 missing data in HC village.

Majority of the respondents took at least one action against malaria in both villages. However, type of personal protective measures and/or mosquito control measures varied considerably among respondents. Using an ITN (92.0% in both villages) was the most frequently reported preventive measures, followed by wearing long sleeves and trousers (49.0% in the VMW village and 43.4% in the HC village).

### Malaria treatment-seeking behavior

The self-reported prevalence of fever within 30 days of the survey was 71.9% in the VMW village and 79.2% in the HC village. Table 3 shows similarities and differences in malaria treatment-seeking behavior between the VMW village and the HC village. In both villages, most fever patients (>90%) sought treatment outside the home. In the VMW village, of those who sought treatment outside the home during the most recent fever episode (n = 104), VMWs were the most frequently reported treatment source (40.4%), followed by the HC (27.9%) and a private clinic (20.2%). In the HC village, of those who sought treatment outside the home during the most recent fever episode (n = 124), the HC was the most frequently reported first treatment source (94.4%). Other reported treatment sources included drug sellers, NGO clinics/hospitals, ODHs, private pharmacies, and the provincial hospital.

**Table 3:** Prevalence of fever 30 days prior to the survey and relevant treatment-seeking behavior

**Treatment -seeking behavior for malaria**	**VMW village**		**HC village**		
	**n**	**%**	**n**	**%**	**p**
Patients who sought treatment outside home*	**n=110**		**n=126**		0.101
Yes	104	94.6	124	98.4	
No	6	5.5	2	1.6	
Treatment source*	**n=104**		**n=124**		<.0001
VMW	42	40.4	0	0	
HC	29	27.9	117	94.4	
Private clinic	21	20.2	4	3.2	
Drug seller	5	4.8	1	0.8	
Other	7	6.7	2	1.6	
Blood test for malaria*	**n=104**		**n=124**		0.570
Yes, Dipstick/Sldie	88	84.6	98	79.0	
Not tested	15	14.4	24	19.4	
Don’t know	1	1.0	2	1.6	
Place to get the blood test*	**n=88**		**n=98**		<.0001
VMW	38	43.2	0	0	
HC	28	31.8	93	94.9	
Private clinic	18	20.5	3	3.1	
Other	4	4.6	2	2.0	
Result of the blood test*	**n=88**		**n=98**		1.000
Positive	70	79.6	78	79.6	
Negative	17	19.3	19	19.4	
Don’t know/don’t remember	1	1.1	1	1.0	
**Time to receive first treatment†**	**n=104**		**n=124**		<.0001
Same day	24	23.1	13	10.5	
Next day	35	33.7	80	64.5	
2 days after the illness started	31	29.8	23	18.6	
3 or more days after the illness started	14	13.5	8	6.5	
**Antimalarial drugs†**	**n=104**		**n=124**		0.032
Yes	98	94.2	106	85.5	
No	6	5.8	18	14.5	
Type of antimalarial taken*	**n=98**		**n=106**		0.555
A+M	25	25.5	26	24.5	
Malarine	14	14.3	14	13.2	
Antimalalrial drugs not recommended	8	8.2	4	3.8	
Paracetamol	7	7.1	5	4.7	
Don’t know/don’t remember	44	44.9	57	53.8	

*Fisher’s exact test.

†Chi-square test.

Majority of the patients with malaria-like symptoms were biologically tested in both villages. In the VMW village, of the 104 patients who sought outside treatment, about 85% were biologically tested for malaria. In the HC village, of the 124 patients who sought outside treatment, 79% were biologically tested for malaria. In the VMW village, of 88 patients who had their blood tested, VMWs were the most frequently reported source of a biological diagnosis (43.2%), followed by the HC (31.8%) and a private clinic (20.5%). In the HC village, of 98 patients who had their blood tested, more than 90% reported having had a blood test at the HC, followed by a private clinic (3.1%) and an ODH (2.0%). In both villages, nearly 80% tested positive and about 19% tested negative.

A bivariate analysis showed a significant difference in the distribution of time before first malaria treatment between the two villages. In the VMW village, of 104 patients who sought outside treatment, 23.1% reported having received the first treatment on the same day as the fever onset. This was higher than the 10.5% in the HC village. In the VMW village, 33.7% received their first treatment on the day after the fever onset, compared to 64.5% in the HC village. In the VMW village, 43.3% received their first treatment two or more days after the fever onset, compared to about 25% in the HC village.

Most of the patients with malaria-like symptoms reported having been treated with antimalarial drug in both villages (>80%). However, nearly half of them could not recall the name of the antimalarial taken. Of those who could recall the name of the antimalarial drug taken, A + M was the primary antimalarial drug taken, followed by Malarine in both villages.

### Comparison of community knowledge about malaria, actions, and malaria treatment between the VMW village and the HC village

Multiple regression analyses demonstrated that the villagers perceived the VMWs’ services equally as good as those provided by the health center (Table 4). After controlling for confounding factors, the following indicators did not show any statistical significance between two villages: community members’ knowledge about malaria transmission (AOR = 0.60, 95% CI = 0.30-1.22) and government-recommended antimalarial (AOR = 0.55, 95% CI = 0.25-1.23), preventive and control measures taken by respondents (Beta = −0.191, p = 0.315), and time before the first treatment (Beta = 0.053, p = 0.721). However, knowledge about malaria symptoms was significantly lower in the village with VMWs than the village with a health center (AOR = 0.40, 95% CI = 0.19-0.83).

**Table 4:** Comparison of community knowledge, action, and malaria treatment between the VMW and the HC villages

		**Adjusted Odds Ratio**	**95%CI**		**β**	**p**
			**Lower**	**Upper**		
Knowledge about the cause of malalria*	VMW(n=153)	0.60	0.30	1.22		
	HC(n=158)					
Knowledge about malalria symptoms*	VMW(n=153)	0.40	0.19	0.83		
	HC(n=159)					
Knowledge about government-recommended drug*	VMW(n=153)	0.55	0.25	1.23		
	HC(n=159)					
Preventive and control measures taken†	VMW(n=110)				-0.191	0.315
	HC(n=124)					
Time before first treatment†	VMW(n=110)				0.053	0.721
	HC(n=124)					

*Multiple logistic regression and †multiple regression, controlling for potential confounders (age, sex, marital status, education, occupation, the number of household member, income, first treatment source, distance from health facility).

## Discussion

The most important finding of this study was that villagers perceived VMWs’ services equally as good as those provided by the health center in receiving malaria diagnosis and effective treatment. This is supported by the evidence that no significant differences were detected in community knowledge about malaria transmission and government-recommended antimalarial, varieties of preventive measures taken, and time before first malaria treatment between the VMW village and the HC village. Even in a remote and resource-poor village without public health facilities, VMWs contributed to provide effective antimalarial treatment with biological confirmation and to disseminate accurate information about malaria to community members.

The two villages however, showed different levels of knowledge on malaria symptoms. Community member’s knowledge about malaria symptoms was significantly lower in the VMW village than that in the HC village. Nevertheless, more than one third could not answer all of three major malaria symptoms in the HC village as well. Due to the absence of the standard definition in assessing level and correctness of knowledge, it is difficult to directly compare our findings with other study findings. However, when just focusing on knowledge on fever as the prime malaria symptoms, community members in both villages were better informed than those in other studies [13,14].

Early recognition of malaria symptoms is the first important step for prompting treatment-seeking behavior. VMWs, as well as the HC staff, need to enhance community knowledge about malaria symptoms to improve access to timely and appropriate treatment before becoming severe.

Unlike knowledge on malaria symptoms, the VMW village and the HC village showed similar levels of knowledge on malaria transmission. Our finding shows that most respondents in both villages were aware that malaria is transmitted through mosquito bites. This is in line with previous studies, which showed high level of knowledge on cause of malaria among community members [13,14]. Nevertheless more than 30% did not know that mosquito bites are the only route for malaria transmission in both villages. The similar result was observed in Tanzania, where only 35% of the respondents believed that mosquito alone are responsible for malaria [15]. As observed in rural Ethiopia, many had misconceptions regarding aetiology of malaria such as lack of hygiene, although they recognized role of mosquitoes in the malaria transmission cycle [16]. VMWs and the health center could make greater efforts to help the community understand the causal connection between mosquito bites and malaria transmission.

Although malaria in pregnancy has devastating effects not only on mothers but also on newborns and infants, pregnant women were not widely perceived as a group at high risk of malaria infection in both villages [17]. While more than 70% of respondents from each village understood that children under 5 are susceptible to malaria infection, only about half of them knew that pregnant women are also at high risk. A recent systematic review of qualitative research demonstrated perceived vulnerability of malaria in pregnancy is specific to the local context [18]. While many studies have demonstrated that pregnant women are particularly vulnerable to malaria infection, their vulnerability is often underestimated as observed in this study, which can place them at even higher risk of infection. Maternal infection in low malaria transmission areas is more likely to result in symptoms, severe disease, and death of the mother or foetus than in high transmission areas [17]. To improve maternal, neonatal and child health in the community, it is crucial that community members understand vulnerability of pregnant women and adverse effects of maternal infection on birth outcomes.

In addition, a large number of respondents could not give the correct name of the government-recommended antimalarial drug in both villages. This result is discordant with the findings observed in other study, where more than 80% of the respondents had knowledge about the names of currently used drug [19]. During the interviews, many respondents tend to recall the name of commercialized antimalarial (Malarine), even though they took antimalarial recommended by government (A + M). This is because Malarine can easily be associated with malaria. Labelling drug packages with user-friendly name such as Malarine may help community members understand and use recommended antimalarial drug.

Various malaria preventive measures were taken in both VMW and HC villages. However, many of those measures seem to be not directly effective or environmentally harmful— such as using insecticide spray, using conventional mosquito nets, and drinking clean/boiled water. This may be partly explained by the fact that VMWs’ training has been less focused on prevention and vector control than diagnosis and treatment [12]. Another possible explanation is that many of community members did not fully recognize mosquito bites as the only cause of malaria transmission. Evidence from Côte d’Ivoire reported that perceived cost was related to the use of preventive measures [13]. Since community members are motivated to take preventive actions, well-organized educational campaigns may help them take cost-effective, environmentally friendly, and sustainable malaria preventive measures such as draining stagnant water and alternating wet/dry irrigation in rice cultivation.

The vast majority (> 90%) sought treatment outside home in both villages. Unlike evidence from northern areas of Pakistan, home remedies and traditional healers were uncommon as first treatment source [20]. In this study, VMWs were a primary treatment source for malaria-like symptoms in the VMW village. Our findings showed that 40% of fever cases were first treated by VMWs, even though the health center is assumed to have better disease management capacity. This may be due to better accessibility of VMWs compared to the health center. Our result is consistent with the finding of the study conducted in Uganda, which also reported the same rate of CHW’s utilisation in case of fever in the preceding month [21]. On the other hand, another study reported much lower rate (24%) of CHW’s utilization in case of febrile illness [4].

Recently CHWs are increasingly called on to manage treatment of not only malaria but also other non-malaria febrile illnesses notably pneumonia and other cause of child mortality [1]. This strategy, known as integrated community case management (iCCM) of childhood illness, may increase the use of VMWs as observed in Nepal [22]. In Cambodia, VMWs’ disease management capacity has been expanded to treat Acute Respiratory Infections (ARI) and diarrhea since 2009. A recent study showed that the quality of VMWs’ services was maintained high even after the scale-up [23]. Future research is needed to evaluate the impact of the scale-up of the programme on the utilization of VMWs, as well as on morbidity and mortality of diarrhea and ARI.

Furthermore, in this study, the elapsed time before seeking the first malaria treatment was long. The two villages did not differ significantly in this regard. While about 57% in the VMW village and 75% received treatment within one day of the onset of symptoms, many waited until two or more days after the illness onset before seeking treatment, which could increase the probability of developing complications. Our finding, however, still encouraging compared to the results observed elsewhere [4,24]. Since VMWs and the health center were the most frequently reported first line of contact in case of fever, they should emphasize the importance of early diagnosis and prompt treatment with effective antimalarials within 24 hours of symptom onset, to avoid complications.

This study had three main limitations. First, it used self-reported data, which are susceptible to reporting bias. However, attempts were made to minimize this potential bias by pre-testing the questionnaire, training interviewers, and making the wording culturally appropriate. Second, the study was not able to explore cause-effect relationships because of the cross-sectional study design. It, thus, could not address the effectiveness of VMW services in improving malaria treatment, community knowledge, and preventive measures. Third, only one VMW village and one HC village were surveyed in this study. Further research with better sampling method and increased sample size should be conducted in other malaria endemic areas in Cambodia to confirm our findings.

Nevertheless, this study provides important information on the quality of VMWs’ services by assessing knowledge, action, and treatment-seeking behavior for malaria among community residents, who are consumers of their services. This information will be useful for improving malaria case management at community level, especially among marginalized populations in underserved remote areas.

## Conclusions

VMWs successfully played an equivalent role as the health center in promoting malaria knowledge and action even in a resource-poor village without health center services. Although VMWs need to make greater efforts to enhance community knowledge about malaria symptoms, this study confirms that the current government policy on VMWs is reasonable and should be expanded to other villages, where malaria still remains a public health challenge.

## Competing interests

The authors declare that they have no competing interests.

## Authors' contributions

SL conceived the study, prepared the project proposal, developed the questionnaire, collected and analyzed data, and wrote the manuscript. JY contributed to the study design, data interpretation, and improvement of the manuscript. KCP reviewed and improved the manuscript. PL and CN trained surveyors and supervised fieldwork. MJ monitored progress of the study and provided guidance during fieldwork and the writing of the manuscript. All authors read and approved the final manuscript.
